# Structural Priming and Inverse Preference Effects in L2 Grammaticality Judgment and Production of English Relative Clauses

**DOI:** 10.3389/fpsyg.2022.845691

**Published:** 2022-06-22

**Authors:** Ran Wei, Sun-A Kim, Jeong-Ah Shin

**Affiliations:** ^1^School of Foreign Studies, Jiangsu Normal University, Xuzhou, China; ^2^Department of Chinese and Bilingual Studies, Hong Kong Polytechnic University, Hong Kong, Hong Kong SAR, China; ^3^Department of English and Literature, Dongguk University, Seoul, South Korea

**Keywords:** structural priming, relative clause, sentence processing, inverse preference effects, production, comprehension

## Abstract

This study investigated inverse preference effects in L2 structural priming of English relative clauses and their potential influences on subsequent learning of target structures. One hundred fourteen Chinese learners of English at a low-to-intermediate proficiency level participated in a structural priming experiment with a pretest-posttest design. The experimental group underwent a priming task in which they orally produced syntactic structures immediately after viewing English object or passive relative clauses as primes, whereas the control group only read sentences unrelated to English relative clauses. A grammaticality judgment task and a sentence completion task were used to measure the inverse preference effect and its subsequent effects on L2 learning. The results showed the presence of structural priming and inverse preference effects in immediate production, which extended to subsequent learning of L2. In subsequent grammaticality judgments and production, L2 learners performed better with English object relative clauses than with English passive relative clauses in comparison with the pretest. The results are discussed in terms of the structural frequency in both L1 and L2 as well as the implicit learning mechanisms of structural priming.

## Introduction

Structural priming refers to a phenomenon whereby a speaker tends to repeat a syntactic structure which was heard or produced in recent discourse (Bock, 1986). For instance, a speaker is likely to produce an English prepositional dative (e.g., *the nanny gave an apple to the boy*) after the same construction was spoken by either the speaker him/herself or an interlocutor in the preceding utterance, even though its alternative construction, double-object dative (e.g., *the nanny gave the boy an apple*), can express an identical meaning. Simply, when the subsequent spoken construction is consistent with a previously produced or heard construction, we can say priming has occurred. The appearance of structural priming is independent of specific linguistic features, so it reflects an abstract syntactic representation constructed in language processing and provides support for structural representation and language acquisition (e.g., Ferreira and Bock, 2006; Pickering and Ferreira, 2008).

While structural priming is well documented in first language (L1) processing and acquisition, there still remains an open question about to what extent second language (L2) structural priming shapes L2 language production and ultimately facilitates language learning. Particularly, L2 structural priming is more intriguing under the phenomena called *inverse preference effect*s. The inverse preference effect means that a less preferred or less common construction is more likely to be primed than its alternative counterpart (i.e., a more preferred or more common structure). Previous studies have shown that L1 speakers are more likely to produce constructions with greater structural flexibility than L2 speakers, and L2 speakers tend to choose relatively preferred structures (e.g., English actives) rather than less preferred structures (e.g., English passives) due to their lower proficiencies in the L2 (e.g., Konopka et al., 2018). In other words, if L2 priming leads to L2 production and relatedly L2 learning, the next question is whether L2 priming effects would be modulated by structural frequency in their L1, L2 or both. Inverse preference effects are closely related to construction frequency in the languages, so L2 inverse preference effects are more complex and dynamic in that structural frequency in L1 and/or L2 and L2 proficiency levels need to be considered.

In this context, the current study aimed to examine whether the inverse preference effect exists in L2 sentence production and facilitates subsequent L2 learning of less preferred constructions. At the same time, this study targeted at complex structures in L2 (i.e., English relative clauses) that strikingly differ from participants’ L1. The evidence for inverse preference effects in structural priming has been observed with mostly simple structures in L1 production (e.g., Bock and Loebell, 1990; Hartsuiker and Kolk, 1998; Hartsuiker et al., 1999; Scheepers, 2003; Bernolet and Hartsuiker, 2010; Segaert et al., 2016), but little research has examined the question of whether it is also apparent in complex structures^1^ in L2 production and extends to the subsequent learning process. Therefore, we compared two complex alternative constructions of English relative clauses (object relative clauses vs. passive relative clauses) to explore whether the inverse preference effect would expand in L2 priming and subsequent learning and is driven by structural frequency in their L1, L2 or both. The findings would expand our understanding on the relationship between structural priming and L2 learning.

## Previous Studies

### Structural Priming as Implicit Learning

Since Bock’s (1986) seminal work in language production, there has been growing attention on structural priming as evidence for abstract syntactic representations and cognitive mechanisms of language processing (see Pickering and Ferreira, 2008 for a review). Over the past three decades, researchers have reached a consensus that priming occurs due to features of the sentence form itself, independent of any other features such as specific lexical items, closed-class elements, thematic roles, and phonology (Bock, 1986, 1989; Bock and Loebell, 1990; Cleland and Pickering, 2003). The occurrence of structure priming has been found from experimental studies across different types of languages (e.g., English, German, Dutch, Chinese, and Russian) and on constructions such as datives, passives, transitives, locatives, genitives, and relative clause attachment in both written and spoken modalities among children and adult speakers (see Mahowald et al., 2016 for a review). Furthermore, corpus-based research has also presented strong evidence for the occurrence of structural priming in naturalistic settings (Gries, 2005, 2011; Gries and Kootstra, 2017).

The frequent occurrence of structural priming across languages and structures in various settings raises the question of the underlying mechanisms. Two major theoretical accounts for its occurrence have been put forward to examine the underlying cognitive mechanisms responsible for structural priming: residual activation and implicit learning. L1 researchers have speculated that priming might occur due to residual activation of lexical-syntactic information in processing, which facilitates the successive production of the target (Dell, 1986; Bock, 1989; Pickering and Branigan, 1998). L1 and L2 comprehension priming studies have provided evidence for this lexical activation account (e.g., Tooley and Bock, 2014; Wei et al., 2017). This activation account attributes the mechanism responsible for structural priming to the transient activation of the surface structure of the prime, which provides a reasonable explanation for the lexical boost effect (in which more target structures are prone to be produced when the prime and the target share identical lexical features such as verbs and nouns). Although the lexical activation account can readily explain short-term priming effects, it cannot adequately explain long-term priming effects. Hence, the error-based implicit learning account (Chang et al., 2006; Jaeger and Snider, 2013; Dell and Chang, 2014) has been posited, whereby structural priming occurs when speakers implicitly make predictions about the upcoming input but fail to meet their prediction (i.e., when they experience a prediction error). These prediction errors in turn allow them to adjust their implicit knowledge, which may facilitate long-term acquisition of the construction. Structural priming is therefore by nature a form of implicit learning.

Previous studies thus far have provided strong evidence that structural priming manifests this implicit learning mechanism. Studies have shown that priming effects are long-lasting even when primes and targets are interrupted by several intervening sentences with a variety of syntactic structures (Bock and Kroch, 1989; Bock and Griffin, 2000; Bock et al., 2007). For example, Bock and Griffin (2000) found priming effects even with ten fillers between each prime and target. Also, priming effects were persistent in a cumulative manner with an increase in the production of target structures from baseline to post-priming sessions (Kaschak and Borreggine, 2008). Furthermore, the cumulative priming effect, in both prime-target pairs and prime-round-target-round^2^ pairs, endured for a day or even several weeks (Kaschak et al., 2011, 2014; Branigan and Messenger, 2016).

In addition to production, cumulative priming effects have also been found in comprehension (e.g., Chang et al., 2006; Fine et al., 2013; Jaeger and Snider, 2013; Fine and Jaeger, 2016; Tooley and Traxler, 2018) and grammaticality judgment (e.g., Luka and Barsalou, 2005; Shin and Christianson, 2012), showing that the recently encountered syntactic structures increased comprehension accuracy and speed as well as grammaticality ratings. In particular, Luka and Barsalou (2005) found that participants rated sentences as more grammatical if they had encountered similar structures earlier, and repeated exposures enhanced the structural priming effect. Shin and Christianson (2012) also found marginal improvement in grammaticality judgment tests following the structural priming session. With regard to the impact of structural priming on L2 comprehension, Kim and McDonough (2016) found the persistent priming effect (i.e., 2 weeks later) in maintaining L2 learners’ comprehension knowledge of English passives. These results provide evidence that implicit learning mechanisms hold for both language production and comprehension.

### Inverse Preference Effects

Further evidence in support of an implicit learning mechanism behind structural priming comes from the inverse preference effect. This refers to the tendency whereby a less preferred or less frequent construction is more likely to be primed than its alternative preferred construction. That is, an inverse relationship exists between structure preference and the magnitude of the priming effect.

The inverse preference effect has been widely found for a variety of structures and languages. In English, Bock (1986) found a greater priming effect for the less frequent passive structure than for the more frequent active structure. Similar frequency biases were also found in Bock and Loebell (1990), Hartsuiker and Kolk (1998), and Hartsuiker et al. (1999). Hartsuiker et al. (1999), for example, investigated L1 Dutch speakers’ structural priming of locatives in sentence initial and sentence final positions. Despite the preference for sentence final locatives at baseline, the priming effect occurred in sentence-initial rather than sentence-final position. Similarly, Bernolet and Hartsuiker (2010) showed that the magnitude of priming effects was greater for double-object datives (low frequency in Dutch) than prepositional datives (high frequency in Dutch). In addition, structural priming was an inverse preference pattern in German datives (Segaert et al., 2016) and relative clause attachment in German (Scheepers, 2003). The inverse preference effect is manifest with the same construction in a less preferred context than in a preferred context (Ferreira, 2003) and among L2 speakers (McDonough and Fulga, 2015).

Apart from strong evidence for inverse preference effects across structures and languages, previous studies have also shown that it could indeed be long-lasting. Hartsuiker and Westenberg (2000) reported an experiment on the priming of word order for auxiliary verbs and past participles in L1 Dutch subordinate clauses. Interestingly, it was found that word order frequency was altered after repeated exposure to primes. The participle-final word order was frequently used at the baseline, but the auxiliary-final word order became relatively more preferred after the experiment, indicating an overall diminishing preference of the participle-final word order over the course of the experiment. A similar inverse preference effect for long-term priming was also found for datives and passives (Hartsuiker and Kolk, 1998) and locatives (Hartsuiker et al., 1999).

L2 priming studies also explored the long-term persistent/cumulative effect of inverse preference using a range of constructions. For example, Kim and McDonough (2016) found that the exposure to passive primes facilitated long-term subsequent production of English passives (i.e., 2 weeks later). Kaan and Chun’s (2018) study also showed that cumulative priming effect was stronger for the less frequent structure (i.e., double object datives for L2 English learners). In Jackson and Ruf’s (2018) study of L1 English learners of L2 German, fronted locative phrases (e.g., *in the kitchen the grandpa drinks hot chocolate*; here in English translation only) were more difficult than non-fronted locative phrases (e.g., *the grandpa drinks hot chocolate in the kitchen*) for L2 German learners who are accustomed to relying on subject-first word order (i.e., SVX) rather than adverb-first word order (i.e., XVS), despite the fact that in German fronted locative phrases appear more frequently than non-fronted locative phrases. There were also long-lasting priming effects for the less preferred phrases by the L2 learners (i.e., fronted locative phrases).

### Structural Frequency in L2 Priming

Inspired by the implicit learning account, L2 researchers have begun to examine the occurrence and effectiveness of structural priming in L2 learning. McDonough and colleagues conducted a series of laboratory-based and classroom-based studies to investigate structural priming effects through interaction and collaboration in L2 development. Their results have demonstrated the facilitative role of structural priming in subsequent L2 production across various constructions such as datives (McDonough, 2006), passives (Kim and McDonough, 2008), *wh*-questions (McDonough and Mackey, 2008; McDonough and Chaikitmongkol, 2010), and adverbial/relative clauses (McDonough et al., 2015). With the pretest-posttest-delayed posttest design, many research findings from L2 priming studies have also confirmed the persistence of priming effects (e.g., McDonough and Mackey, 2008; McDonough and Chaikitmongkol, 2010; Shin and Christianson, 2012).

While the relationship between structural priming and L2 learning is well documented using a variety of constructions, there still remain open questions about whether and how structural frequency modulates L2 priming magnitude and duration. Jackson and Ruf (2018) found the long-term priming effect for sentences with less preferred fronted locative phrases in the case of L1 English–L2 German speakers, but it was not supported by L1 German–L2 English speakers in Jackson and Hopp (2020). This inconsistency is likely due to the structural frequency in L1 and/or L2. Shin and Christianson (2012) suggests that L2 priming is modulated by structural frequency (see also Flett et al., 2013; Kaan and Chun, 2018; Montero-Melis and Jaeger, 2020). For proficient L2 learners, L2 frequency plays a modulated role in L2 priming, but for beginning or intermediate L2 learners L1 frequency of alternative structures has an impact on the priming effect. Nevertheless, targeting at less proficient L2 learners, Jackson and Ruf (2017) only found long-term priming effects with fronted temporal phrases rather than fronted locative phrases, even though both of them are L1-preferred adverbial-first structures. It is therefore still uncertain about whether L2 learners’ structural preference is modulated by their L1, L2 or both, especially for learners at lower proficiency levels.

### Cross-Linguistic Difference Between English and Chinese ORCs/PRCs

English object relative clauses (ORCs; e.g., *Here is the reporter that the senator saw*) and passive relative clauses (PRCs; e.g., *Here is the reporter that was seen by the senator*) have the head noun fixed as the antecedent noun. Therefore, even though structure choices between simple active/passive transitive sentences (2a-2b) vary with the order of the agent and patient nouns, there is no change of noun order in complex English ORCs and PRCs (1a-1b).

1a. English ORC: *Here is the reporter that the senator saw*.
1b. English PRC: *Here is the reporter that was seen by the senator*.
2a. Active Transitive: *The senator saw the reporter*.
2b. Passive Transitive: *The reporter was seen by the senator*.

On the other hand, in the English ORC structure, the antecedent noun is the object in the relative clause, while in the English PRC structure, the antecedent is the subject in the clause. These target structures can alternate with each other in syntactic structures but express similar semantic meaning, and the feature of structural alternation is a prerequisite for using a structural priming paradigm to explore inverse preference effects (Bock, 1986; McDonough and Trofimovich, 2009). In structural similarity, both ORC and PRC structures have inter-clausal elements at the upper hierarchy of accessibility (Keenan and Comrie, 1977), which deepens the difficulty in language processing and learning.

Cross-linguistically, English and Chinese relative clauses are quite distinct. First of all, Chinese relative clauses are head-final, whereas English relative clauses are head-initial. In Chinese, the head noun of a relative clause (underlined in the example sentences below) is at the end of the clause, and the difference between Chinese ORCs and PRCs is the passive marker “bèi (被)” added before the embedded noun in PRCs. For example, a Chinese ORC (e.g., cānyìyuán kànjiàn de nàgè jìzhě, 参议员看见的那个记者; *“*the reporter that the senator saw”) can be altered to a Chinese PRC (**bèi** cānyìyuán kànjiàn de nàgè jìzhě, **被**参议员看见的那个记者; “the reporter that was seen by the senator”) by putting in the passive marker “bèi (被).” In English, however, the head noun of a relative clause is head-initial and is followed by the clause, and English ORCs and PRCs differ in the distance between a filler and a gap. The distance between the filler and the gap is long in English ORCs (e.g., *the senator* *_filler_ that the reporter attacked ^_gap_*), while it is shorter in English PRCs (e.g., *the senator* *_filler_ that ^_gap_ was attacked by the reporter*). Due to these differences between Chinese and English, even after the quite lengthy formal instruction of English from elementary until high school, Chinese learners still struggle with accurately using English relative clauses, especially in oral production (Schachter, 1974).

**Figure 1:**
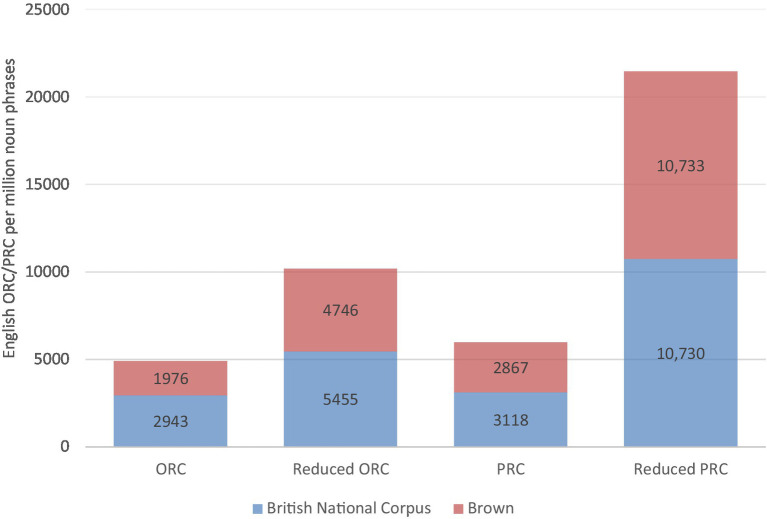
Distribution of English ORCs/PRCs in British National Corpus and Brown Corpus (per million noun phrases) based on Roland et al. (2007).

Comparing English ORCs and PRCs, previous L1 studies showed that ORCs are more difficult than their PRC alternatives (Gennari and MacDonald, 2008) because an inequality in cognitive burden on working memory leads to a discrepancy in processing difficulty; the longer distance between the filler and the gap in English ORCs entails a greater cognitive burden and thus becomes more difficult in processing and learning in comparison to PRCs. Concerning L1 production, English PRCs are more likely to be produced than ORCs in both experimental environments (Gennari and MacDonald, 2009; Gennari et al., 2012) and naturalistic settings (Roland et al., 2007). Figure 1 exhibits a structural bias toward English PRCs in two major corpora.^3^

In sum, the ORCs and PRCs of English and Chinese are strikingly different (L1 Chinese: head-final relative clauses vs. L2 English: head-initial relative clauses). Thus, for L1 Chinese learners of English, there are no L1 Chinese equivalents to L2 English ORCs and PRCs. On the contrary, from the perspective of structural frequency within English, ORCs are less preferred than PRCs, especially when relatives are with animate heads.

### Research Gaps

Previous L1 priming studies have indicated that the magnitude of priming effects for less preferred structures is greater than for alternative preferred structures and its effect of structural bias could be lasting because a weaker expectation for less preferred structures leads to a larger prediction error and greater alterations to the knowledge system. However, it is still not clear how the persistence of inverse preference effects shown in L1 processing would expand to L2 learning. Considering inverse preference effects depend on speakers’ familiarity with construction frequency in the languages that the speakers know, inverse preference effects in L2 processing are more complicated since two languages (L1 and L2) are always involved and L2 proficiency varies inevitably.

Also, literature on the extension of inverse preference effects in L2 learners’ subsequent performance is scarce, and thus more empirical evidence is needed from different structures and languages. In addition, complex constructions have received little attention in previous L2 priming studies. L2 learners may still struggle with the use of the complex structures as compared to native speakers, especially in simultaneous oral utterances, even with years of formal grammatical instruction. Furthermore, previous L2 priming studies have predominantly explored priming effects in subsequent L2 production only. To our best knowledge, two studies addressed both subsequent comprehension and production, although they failed to obtain consistent results. For English double-object dative and phrasal verb construction, Shin and Christianson (2012) found marginal overall improvement in grammaticality judgment tests following the structural priming session for L1 Korean-L2 English learners, while Kim and McDonough (2016) managed to show priming effects for both comprehension and production after priming for L1 Korean learners of L2 English. Since both receptive and productive knowledge are crucial in L2 learning, it is necessary to investigate whether priming effects could benefit subsequent receptive grammatical knowledge, to enhance our understanding of the association between the two in L2 learning.

Based on the aforementioned research gaps, this study intended to investigate the impact of inverse preference effects in promoting L2 learners’ subsequent grammaticality judgments and production of English relative clauses (i.e., English ORC and English PRC) after priming treatment. Three corresponding research questions are formulated as follows:

1. Does the inverse preference effect manifest itself in L2 learners’ immediate primed production of English ORCs and PRCs?
2. Does the inverse preference effect influence L2 learners’ subsequent grammaticality judgments of English ORCs and PRCs?
3. Does the inverse preference effect influence L2 learners’ subsequent production of English ORCs and PRCs?

## Materials and Methods

### Participants

Participants were 114 native Chinese learners of English from a provincial university in mainland China. They were all non-English major sophomores with ages ranging from 19 to 22 years old (*Mean* = 20.11, *SD* = 0.76). They had learned English for 11.41 years on average (*SD* = 1.49), and their English proficiency level was deemed to be low-to-intermediate, with a mean score of 17.43 out of 40 (*SD* = 4.12) in the C-test (Schulz, 2006, unpublished doctoral dissertation).^4^ During their involvement in this study, they were enrolled in a compulsory English course for 1.5 h per week, which focused on practicing spoken English in communicative settings, with less attention to grammar. The participants were randomly assigned to two groups, either to an experimental group (*N* = 57) or to a control group (*N* = 57), and were provided CN¥ 50 as compensation for their participation.

### Target Structures

The target structures were English ORCs and PRCs, which were selected to facilitate comparison with previous studies that have focused on simple sentences and explored inverse preference effects in L2 priming and learning (e.g., Kim and McDonough, 2016; Jackson and Ruf, 2018; Kaan and Chun, 2018). Moreover, the learning of English ORC/PRC, especially ORC, has been shown to be obstacles for Chinese learners of L2 English (Schachter, 1974; Gennari and MacDonald, 2008). The exploration of the inverse preference effect of English ORCs vs. PRCs in L2 priming and learning could better our understanding about the effect of structural priming in L2 learning of complex sentence structures.

### Research Design

The present study employed a pretest-posttest design with experimental and control groups to investigate the immediate and cumulative inverse preference effects in Chinese learners’ primed production^5^ and subsequent grammaticality judgments and production of English ORCs and PRCs. Both groups carried out the same grammaticality judgment task (GJT) and sentence completion task (SCT) before and after the main treatment session (priming vs. no priming). In the main session, the experimental group participated in a priming task where learners had to complete sentences with words provided after being exposed to prime prompts with either ORC or PRC structures, while the control group participated in a non-priming task, in which they only read filler sentences unrelated to the target relative clauses without any sentence completion requirements. Group (experimental group and control group) was a between-subjects variable, and prime (ORC and PRC) and phase (pretest, posttest, and delayed posttest) were within-subject variables in the study. The dependent variables were the proportion of ORC and PRC responses produced during the main session as well as accurate judgments and production of ORCs and PRCs in pretests and posttests.

### Procedure

The entire experiment lasted around 85 min for each participant, which was divided into two separate days (Table 1). At the beginning of Day 1, a language background questionnaire was distributed for the participants to fill in their demographic information (5 min), followed by a language proficiency C-test (10 min). Then, the SCT and GJT pretests were conducted (15 min) to test their prior grammatical knowledge of target structures. The SCT preceded the GJT in order to avoid any GJT influence on their SCT performance. Immediately after the pretests, the main session was carried out: the priming task for the experimental group or the non-priming task for the control group (20 min). At the end of Day 1, the SCT and GJT posttests were administered in sequence (15 min). Three days later (Day 4), the participants took part in delayed SCT and GJT posttests (15 min) and then answered to a post-experiment questionnaire (5 min). The post-experiment questionnaire was given to confirm that the participants were not aware of the target syntactic structures repeated during the experiment nor the purpose of the experiment. No participant correctly remembered which structures were repeated nor guessed the purpose of the experiment. E-Prime 2.0 was used to present stimuli and record responses in the pretests, the main session, and the posttests.

**Table 1:** Overview of the experiment.

Day	Session	Task
1	Language Background Questionnaire	Writing about one’s language background
	Proficiency Test	C-Test
	Pretest	SCT + GJT
	Main Session (Priming Taskvs. Non-Priming Task)	Sentence Completion vs. Sentence Reading
	Posttest	SCT + GJT
4	Delayed Posttest	SCT + GJT
	Post-experiment Questionnaire	Reflecting one’s experience during the experiment

### Instruments

#### Grammaticality Judgment Task

A GJT was used to collect data on receptive syntactic knowledge from the pretest and posttest, with the same testing types but different items (Appendix 1). In each test, a total of 20 test items with 16 critical items and four filler items were generated. Of the 16 critical items, four were pairs of grammatical/ungrammatical sentences with ORC structures and another four pairs with PRC structures. Participants were instructed to judge the grammaticality of each sentence with a button press of J (for true) or F (for false) and then to orally explain their reasons for their judgments. The purpose of oral explanations was to confirm that participants’ judgments were fully grounded on their complete understandings of ORCs and PRCs rather than on non-grammatical judgments or some judgments unrelated to syntactic knowledge of ORCs and PRCs. Then, only the structures judged correctly with correct explanations were considered as correct judgment of ORC/PRC. After the explanations, the participants pressed the spacebar to advance to the next trial.

#### Sentence Completion Task

To collect production data from the pretest and posttest, a modified oral SCT was used (see Appendix 2). The SCT was initially adopted by Pickering and Branigan (1998) to elicit written production data and later used in other studies to elicit oral production data as well (e.g., Hartsuiker and Westenberg, 2000). In the present study, some modification was made by providing three additional words as cues, considering the elicitation difficulty of relative clauses for L2 learners. Twelve incomplete sentences beginning with the phrase *Here is* and three words in parentheses [e.g., *Here is____________*. (*attack, senator, reporter*)] were presented on the computer screen. The participants were required to first point out the agent of the sentence they planned to produce and then to orally complete each sentence. A microphone was used to record the utterances of sentences produced. After they had finished, they were prompted to press the spacebar to advance to the next slide.

#### Treatment for the Experimental Group

**Figure 2:**
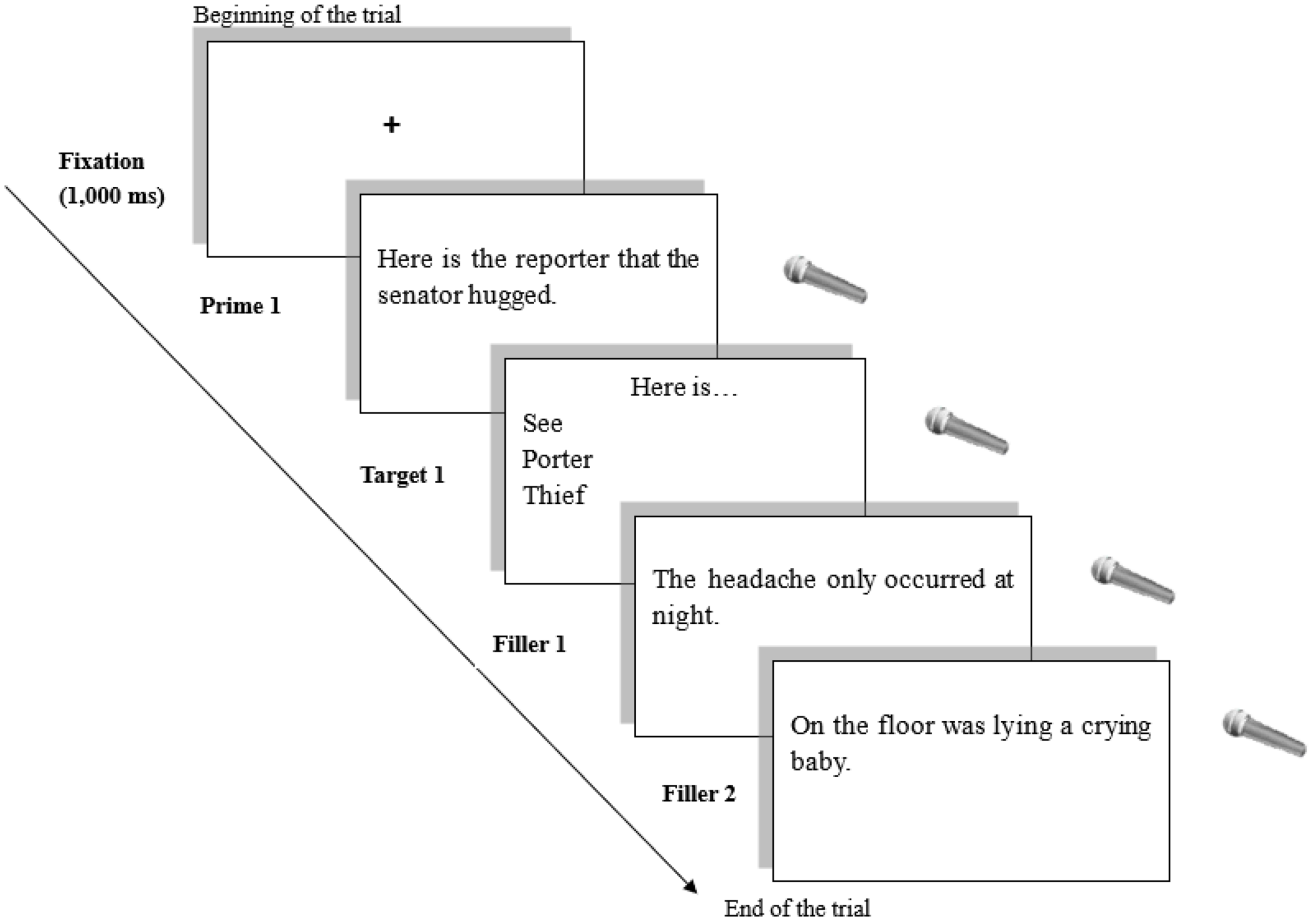
Example of the Main Session for the Experimental Group (Priming Task: Sentence Completion; ORC Prime).

After a brief instruction on the procedure, participants in the experimental group finished four practice items. Then, a fixation cross (+) was shown on the screen for 1,000 ms, which was followed by a prime sentence requiring participants to read aloud and then press the spacebar to advance to the next slide. When an incomplete sentence (i.e., a target) appeared on the screen, they had to orally complete the sentence with the words provided in parentheses as in the pretest (see Figure 2). A rest cue was shown on the screen every 48 sentences to allow for a short break at regular intervals, and the treatment session lasted approximately 20 min.

#### Task for the Control Group

**Figure 3:**
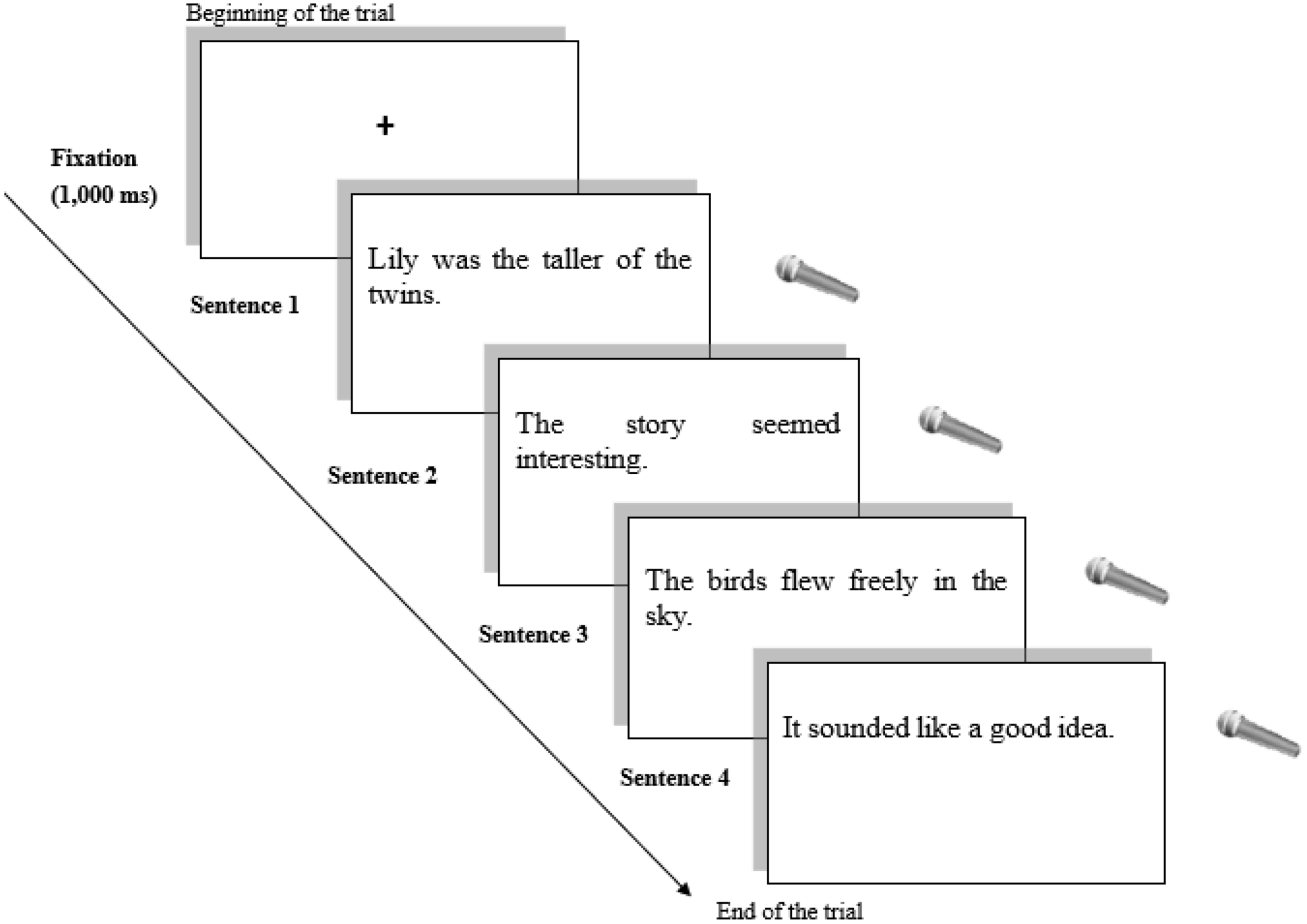
Example of the Main Session for the Control Group (Non-priming Task: Sentence Reading).

A sentence reading task was used for the control group with the same numbers of sentences as those used in the oral sentence completion task for the experimental group. At the very beginning, brief instruction was provided to the participants. After a fixation cross sign (+) for 1,000 ms, they had to read the sentences as quickly as possible, which was audio-recorded (see Figure 3). The break interval was kept the same as the one provided in the priming task, and the lengths and numbers of the sentences used in the task were also identical to those in the priming task.

### Materials for the Treatment Session

For the prime sentences, length was controlled at eight words for ORCs and ten words for PRCs to reduce sentence-length influences on the priming effect. Each set consisted of two priming sentences, an English ORC sentence (e.g., *here is the girl that the boy kissed*) and an English PRC sentence (e.g., *here is the girl that was kissed by the boy*). Each priming sentence is followed by a target-eliciting sentence that initializes with *here is* along with three target-eliciting words, which might induce two expected targets (see Example 1). Examples 1-1a and 1-1b are two conditions of the prime, and Example 1–2 shows the words provided to the participants to induce a target sentence after the prime (see Appendix 4 for the list of target-elicitation words). Examples 1-3a and 1-3b are two expected targets for the given primes. If the priming sentence 1-1a is given, the expected target under such priming condition is 1-3a. Similarly, if the priming sentence 1-1b is provided, 1-3b is the expected target accordingly. Since both ORCs and PRCs exhibit similar semantic consistency, the participants were likely to produce either ORCs or PRCs after the prime prompt. Care was taken to make sure both nouns and verbs used in the prime did not appear again elsewhere.

Example 1

Two Conditions of the Prime

1-1a. Here is the reporter that the senator hugged. (ORC)
1-1b. Here is the reporter that was hugged by the senator. (PRC)

Target-Eliciting Words

1-2. Here is ________________________. (attack, porter, thief)

Two Expected Targets

1-3a. Here is the porter that the thief attacked. (ORC-primed output)
1-3b. Here is the porter that was attacked by the thief. (PRC-primed output)

All 36 experimental prime-expected target pairs were divided into two conditions, with 18 sentences in each condition for counterbalancing, and every participant saw all 36 sentences in total. A Latin-square design was used to eliminate order effects by placing 18 expected targets after ORC primes and the other 18 after PRC primes.

In addition to the critical stimuli, 72 fillers were included in each experimental list to prevent participants from potentially inferring the purpose of the experiment. The filler items were composed of three to twelve words. None of the filler items contained the same verb or structure, so neither the experimental items nor the fillers would lead to any preference for producing either ORC or PRC constructions. Hence, for each list, there were 144 items consisting of 36 prime-expected target pairs and 72 fillers. Prior to the actual experiment, eight practice items were presented for the participants to familiarize themselves with the procedure.

For the experimental group, a total of 36 experimental prime-target sets were constructed as critical stimuli in the study (Appendix 3). Only animate nouns representing human beings without apparent semantic inference^6^ were selected as head nouns of matrix sentences as well as subjects or agents in the relative clauses to exclude animacy influences (Trueswell et al., 1994; Gennari and MacDonald, 2008) and the pronominal effects of embedded nouns (Gordon et al., 2001; Warren and Gibson, 2002; Reali and Christiansen, 2007) in relative clause processing. For predicates, only transitive verbs representing actual behavior were chosen, and the relative complementizer was constrained to use of the relative pronoun *that* rather than *who* or *which* to exclude lexical intervention effects. The relative pronoun *that* can refer to either animate or inanimate noun phrases, but other relativizers such as *who*, *which*, *what* can only refer to either animate or inanimate noun phrases. According to a corpora study involving Switchboard, Brown and WSJ corpora, less than 1.5% of all ORCs occurred with *who*, but 36–88% of ORCs had *that* (Marcus et al., 1995). In other words, ORCs might be more likely to occur with the relative pronoun *that* compared to other relativizers. All lexical items used in the experiment were taken from the students’ textbooks and checked for frequency in the New General Service List (Browne et al., 2013) to guarantee their familiarity with the provided vocabulary items and to avoid any undesired lexically driven results.

For the control group, the same number of 144 sentences were used in the non-priming sentence reading task, with the 72 filler items used in the experimental group as well as another 72 sentences unrelated to the target structures. The length of sentences was around three to twelve words. None contained the same verbs to ensure that the experimental stimuli would not lead to any preference for the target constructions.

### Scoring and Data Analysis

For the GJT data, accuracy depended on whether participants made correct syntactic judgments on ORC and PRC structures. Every correct judgment of an ORC or PRC structure was coded as 1, and incorrect as 0.

Unlike clear-cut answers for the GJT, the SCT yielded various kinds of responses, and so the following scoring criteria were established. An ORC structure refers to a construction with a head noun followed by the relativizer *that* and a subject noun plus the transitive verb in a relative clause. The PRC structure refers to a construction with a head noun, followed by the relativizer *that* and the complete form of a passive structure (i.e., be + V-ed + by) plus the agent noun in a relative clause. ORC or PRC structures with the relative pronoun *that* (e.g., *here is the singer that the dancer disliked* or *here is the singer that was disliked by the dancer*) were deemed correct target responses because participants were instructed to use the relativizer *that* for producing relative clauses, and all the primes used in the experiment were either ORCs and PRCs. Other responses were not considered, such as reduced ORCs or PRCs (e.g., *here is the singer the dancer disliked* or *here is the singer disliked by the dancer*), subject relative clauses (hereinafter, SRC, e.g., *here is the singer that disliked the dancer*), present participle clauses (verb + ing; e.g., *here is the singer disliking the dancer*), random combinations of noun phrases (e.g., *here is the police and the reporter and the phone*), or ungrammatical structures (e.g., *here is the governor insults the specialist*). However, errors irrelevant to structural features (e.g., spelling mistakes, tense, or aspect) were remained in consideration, because spelling errors aren’t indicative of structural processing and research demonstrates that tense and aspect features do not influence priming (Pickering and Branigan, 1998).

For the priming task, the orally primed productions were transcribed and analyzed by the first author and confirmed by another person, and the inter-rater reliability was near perfect agreement, with a Cohen’s kappa statistic of *k* = 0.998. The scoring criteria for ORCs and PRCs structures were the same as for the SCT. Each correct production of an ORC or PRC structure was coded as 1, and other responses were coded as 0.

All data were statistically analyzed using mixed-effects models using the glmer function in R (R Development Core Team, 2017). Dependent variables were judgment accuracy and production of ORC and PRC structures. The fixed effects predictor (effects-coded −0.5/0.5), and random intercepts for subjects and items, and by-subject and by-item random slopes were included in the model. All models were fit using the maximal random effects structure including main effects and interactions that converged (Barr et al., 2013). If the model did not converge, random slope structure was simplified until convergence was achieved.

## Results

The pretest results were first analyzed to check whether L2 learners showed structural bias (English ORC vs. English PRC sentences) prior to priming tasks, and then, the main priming results were analyzed according to the research questions. In the grammaticality judgment pretest, participants correctly judged the grammaticality of English ORC and PRC types (94.6% of the total responses), and no preference was found between PRCs (62.28%, 568 out of 912 trials) and ORCs (58.22%, 531 out of 912 trials) in judgment accuracy (estimate = 0.396, SE = 0.347, *z* = 1.142, *p* = 0.253). However, in the production pretest, 18.64% of the total responses were English ORCs and PRCs, and L2 learners produced more English PRCs (23%, 158 out of 684 trials) than ORCs (14%, 97 out of 684 trials; estimate = 1.746, SE = 0.265, *z* = 6.587, *p* < 0.001), showing the preference towards English PRCs in L2 production.

### Inverse Preference Effects in Immediate Production in the Priming Session

The first research question concerns the inverse preference effect occurring in immediate production among the experimental group for the priming task of the main session. For this question, it was necessary to first confirm the occurrence of structural priming and then compare the magnitude of priming effects between the two target structures in the primed sentence completion task.

**Figure 4:**
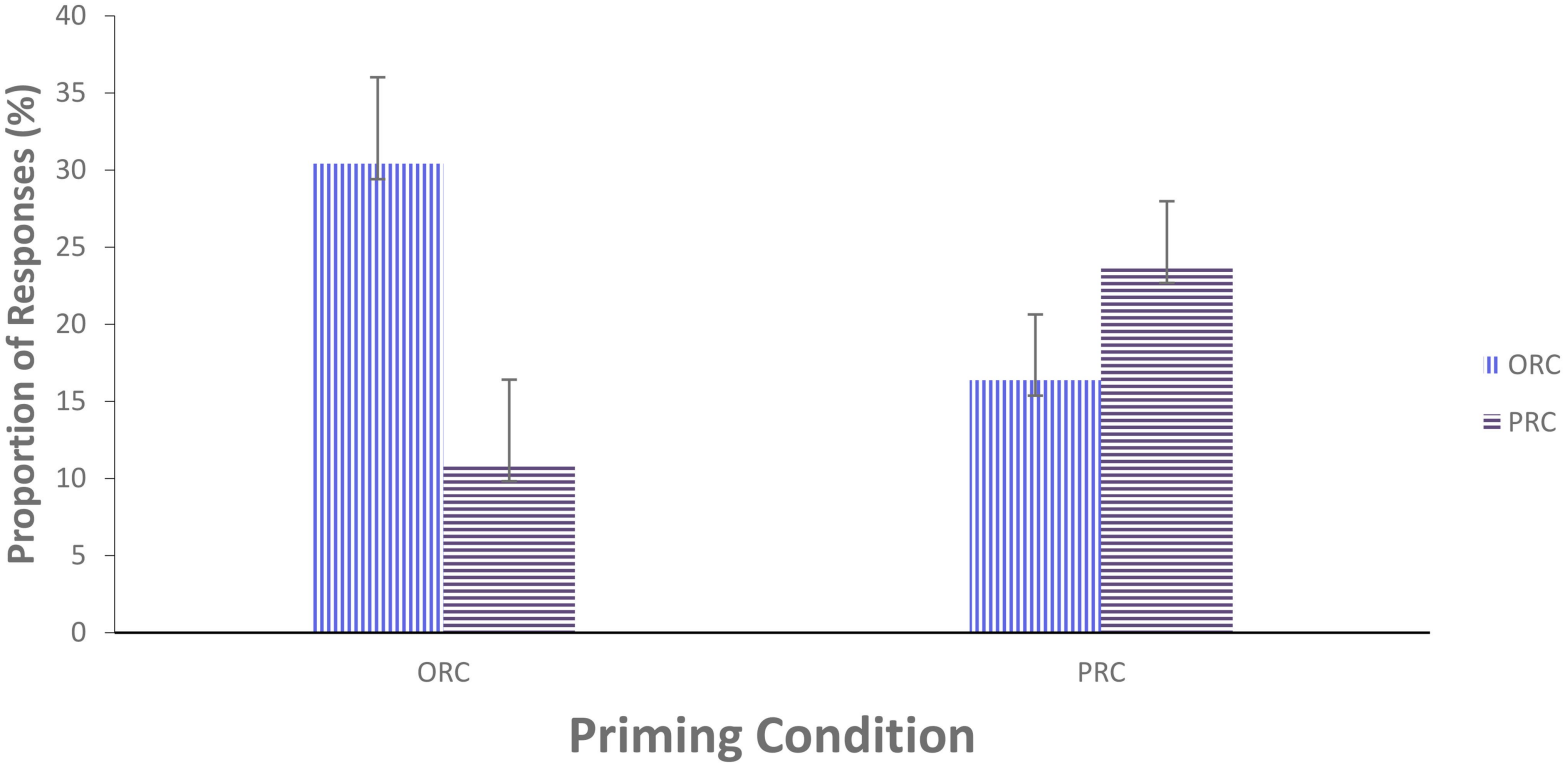
Proportion of Responses in the Priming Conditions. Note. Error bars represent 95% CIs for the difference between participant means.

The target responses produced in the experimental group priming task were 2,052 in total, with eight responses missing or incomplete, and thus, 0.39% of the trials were removed. The percentage of all the types of produced structures during the priming session is shown in Table 2. The proportion of ORC responses was greater than that of PRC responses, even though nearly half of the responses were other types including SRCs, present participle clause, reduced PRCs, ORC/PRC using the word *who*, and ungrammatical structures. As seen in the descriptive data for responses under the two priming conditions in Figure 4, the proportion of ORC responses produced was much higher following ORC prompts (30.41%) than following PRC prompts (10.82%), and likewise, the proportion of PRC responses was higher after PRC prompts (23.68%) than after ORC prompts (16.37%). This indicates that participants were more likely to produce a target structure following corresponding prime structure than following its alternative structure prime; that is, the structural priming effect occurred in the target structures of English ORCs and PRCs. Additionally, the magnitude of priming effects for ORC responses (30.41%) was stronger than for PRC responses (23.68%).

**Table 2:** Percentage (%) of all types of produced sentences in the priming session.

Type	Percentage (%)
ORC	23.48
ORC using the word *who*	0.10
PRC	17.32
PRC using the word *who*	1.08
Reduced PRC	0.98
SRC	24.07
Present Participle Clause	6.02
Ungrammatical Structure	26.96

To statistically verify the descriptive evidence for the occurrence of structural priming and the inverse preference effect in the priming session, a mixed-effects model was run with binary outcome of prime-target repetition (1 vs. 0). The fixed predictors Prime Type (effects coding ORC = −0.5 vs. PRC = 0.5) and Trial Number (i.e., 1–36, there are 36 stimuli of prime-target pairs) were entered into the model. The random effects structure included random intercepts and random slopes for subjects and items. This model revealed a significant main effect of Prime Type, with those primed with the ORC prime prompts demonstrating a higher percentage of prime-target repetition than those primed with the PRC prime prompts (estimate = −0.476, SE = 0.120, *z* = −3.978, *p* < 0.001). There was also a significant main effect of Trial Number, indicating that the percentage of prime-target repetition across both prime types increased over trial numbers (estimate = 0.038, SE = 0.007, *z* = 5.594, *p* < 0.001). This therefore shows the tendency of the inverse preference effect in structural priming; that is, the less preferred structure (ORCs) was more likely to be primed than the preferred structure (PRCs).

### Inverse Preference Effects in Subsequent Grammaticality Judgments

The second research question investigates the facilitative role of structural priming and the inverse preference effect extending to subsequent grammaticality judgments. To address this question, it was necessary to compare the performance of the experimental group and the control group in the GJTs at three phases (pretest, posttest, and delayed posttest) and their increases in judgment accuracy for ORCs and PRCs.

For the GJT of the pretest phase prior to the main session, judgment accuracy of English ORCs was lower for the experimental group (57.02%) than the control group (59.43%), whereas that of English PRCs was higher for the experimental group (66.67%) than the control group (57.89%). To statistically verify the difference between two groups in the pretest, a mixed-effects model was fit with judgment accuracy (1 vs. 0) as the dependent variable. The fixed effects predictors were Group (effects-coded: experimental group = −0.5 vs. control group = 0.5) and Type (effects-coded: ORC = −0.5 vs. PRC = 0.5), while random intercepts and random slopes for subjects and items were also included into the model. There were no statistically significant main effects for Group (estimate = −0.255, SE = 0.163, *z* = −1.564, *p* = 0.118) and Type (estimate = 0.125, SE = 0.157, *z* = 0.797, *p* = 0.425) on judgment accuracy. That is, both groups demonstrated no difference in grammatically judging target ORC and PRC structures prior to the priming session.

**Figure 5:**
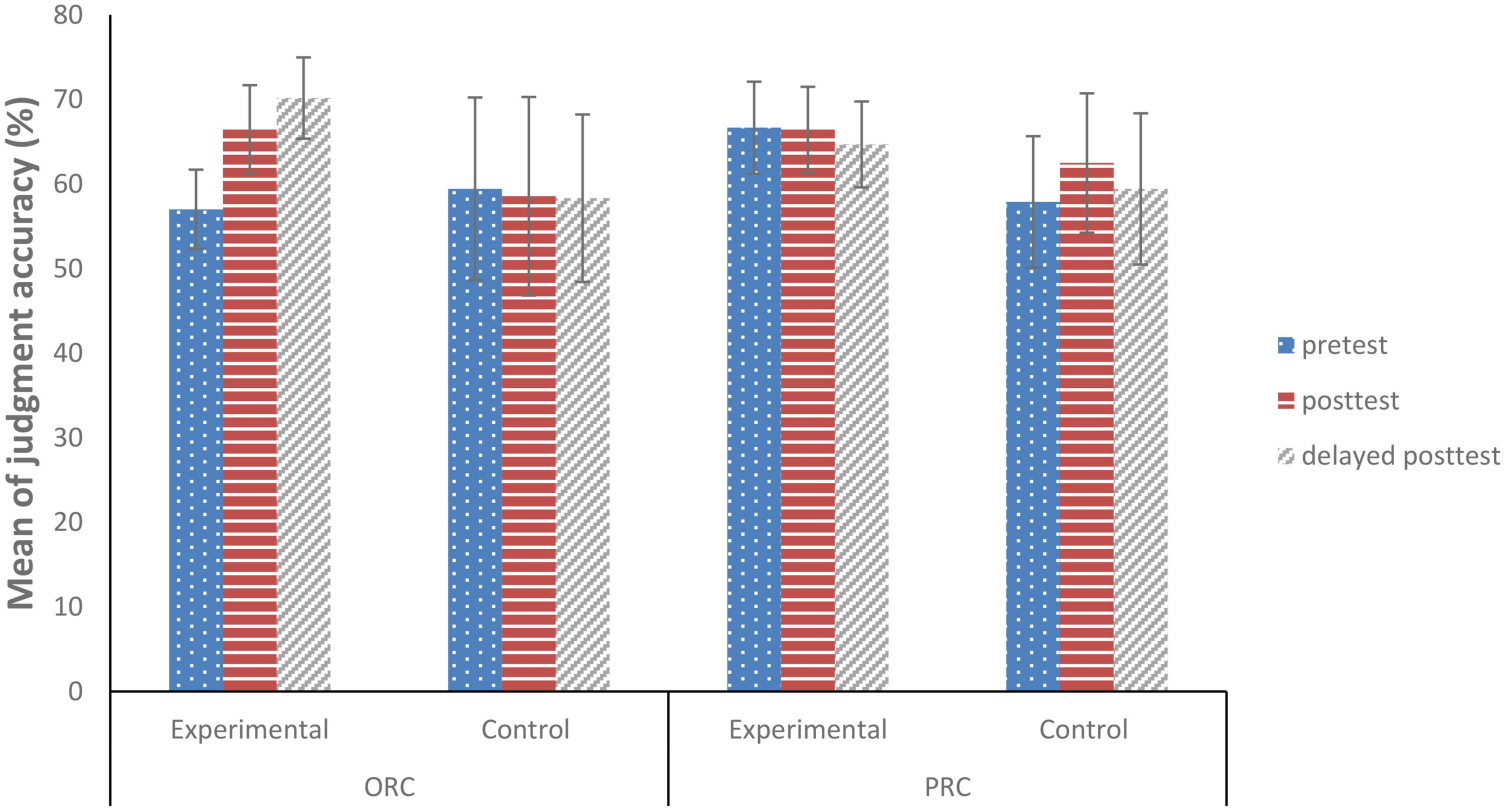
Judgment Accuracy for English ORCs and PRCs at Three Phases. Note. Error bars represent 95% CIs for the difference between participant means.

Figure 5 presents the descriptive results for grammaticality judgment accuracy at pretest, posttest, and delayed posttest for the experimental group and the control group. The experimental group showed an increase in judgment accuracy for ORC structures from pretest (*M* = 57.02%) to posttest (*M* = 66.45%) but a slight change in judgment accuracy for PRC structures from pretest (*M* = 66.67%) to posttest (*M* = 66.45%). However, for the control group, the judgment accuracy for ORC structures changed from pretest (*M* = 59.43%) to posttest (*M* = 58.56%), whereas that for PRC structures changed from pretest (*M* = 57.89%) to posttest (*M* = 62.5%).

To statistically assess the differences among phases and groups, a mixed-effects model was run with judgment accuracy of target structures as a dependent variable (1 vs. 0). Fixed effects included Phase [within-subject factor with effects coding: pretest = −0.5 vs. posttest = 0.5 (the remaining variable = 0) and pretest = −0.5 vs. delayed posttest = 0.5 (the remaining variable = 0)], and Group (between-subject factor with effects coding: experimental group = −0.5 vs. control group = 0.5), and two-way interactions between Phase and Group. The final model included fixed effects for Phase and Group and their interactions, as well as random effects for subject and item intercepts and slopes.

As shown in Table 3, there was a significant effect for the interaction between Group and Phase (pretest vs. delayed posttest) in judgment accuracy for ORC structures (estimate = −0.559, SE = 0.258, *z* = −2.169, *p* = 0.030). For the experimental group, L2 learners increased their judgment accuracy for ORC structures from pretest to delayed posttest after the priming task, and the increase was greater for the experimental group than for the control group. Furthermore, the increase in judgment accuracy was maintained from posttest to delayed posttest verifying the persistence of structural priming effects in receptive knowledge of English ORCs. For PRC structures, there was no significant effect for Phase (pretest vs. posttest; estimate = 0.157, SE = 0.125, *z* = 1.252, *p* = 0.211), Phase (pretest vs. delayed posttest; estimate = −0.103, SE = 0.133, *z* = −0.775, *p* = 0.438) or Group (estimate = −0.229, SE = 0.198, *z* = −1.156, *p* = 0.248) in the grammaticality judgments for PRC structures. L2 learners failed to increase their judgment accuracy for PRC structures through the priming task. Similar evidence came from a statistically non-significant Phase (pretest vs. posttest) by Group (estimate = 0.182, SE = 0.252, *z* = 0.722, *p* = 0.470) or Phase (pretest vs. delayed posttest) by Group (estimate = 0.100, SE = 0.254, *z* = 0.393, *p* = 0.694) interaction effect, indicating that changes in judgment accuracy over three phases did not differ between the two groups.

**Table 3:** Judgment accuracy for ORC and PRC structures at three phases.

	Predictors	Estimate	SE	*z* value	Pr(>|*z*|)
ORC	(intercept)	0.585	0.121	4.828	<0.001^***^
	Group (experimental vs. control)	−0.229	0.198	−1.156	0.248
	Phase (pretest vs. posttest)	0.053	0.122	0.434	0.665
	Phase (pretest vs. delayed posttest)	0.239	0.133	1.805	0.071
	Group: Phase (pretest vs. posttest)	−0.265	0.250	−1.061	0.289
	Group: Phase (pretest vs. delayed posttest)	−0.559	0.258	−2.169	0.030^*^
PRC	(intercept)	0.709	0.124	5.705	<0.001^***^
	Group (experimental vs. control)	−0.279	0.189	−1.480	0.139
	Phase (pretest vs. posttest)	0.157	0.125	1.252	0.211
	Phase (pretest vs. delayed posttest)	−0.103	0.133	−0.775	0.438
	Group: Phase (pretest vs. posttest)	0.182	0.252	0.722	0.470
	Group: Phase (pretest vs. delayed posttest)	0.100	0.254	0.393	0.694

^*^p < 0.05, ^***^p < 0.001; Final model: Accuracy ~ Phase (pretest vs. posttest) * Group + Phase (pretest vs. delayed posttest) * Group + (1|Subject) + (1|Item).

To investigate whether the inverse preference effect extends to subsequent grammaticality judgments, another model was run with judgment accuracy (1 vs. 0) as the dependent variable. Type (ORC = −0.5, PRC = 0.5), Phase [pretest = −0.5 vs. posttest = 0.5 (the remaining variable = 0) and pretest = −0.5 vs. delayed posttest = 0.5 (the remaining variable = 0)] and Group (experimental = −0.5 vs. control = 0.5) were entered as fixed effect predictors as well as their interaction terms. The random effects structure included random intercepts and random slopes for subjects and items. A significant three-way interaction effect was found for Phase (pretest vs. posttest) by Type by Group (estimate = 0.779, SE = 0.299, *z* = 2.603, *p* = 0.009), and for Phase (pretest vs. delayed posttest) by Type by Group (estimate = 0.862, SE = 0.304, *z* = 2.831, *p* = 0.005), suggesting that the increase in judgment accuracy for ORC structures from pretest to posttest and from pretest to delayed posttest was greater than for PRC structures in the experimental group. That is, the inverse preference effect did extend to subsequent grammaticality judgments.

In sum, structural priming promoted L2 learners’ accurate judgments for ORC structures with a long-term priming effect, but failed to promote judgment accuracy for PRC structures. Therefore, the evidence indicates that the inverse preference effect extends to some subsequent grammaticality judgments.

### Inverse Preference Effects in Subsequent Production

To address the third research question, it was necessary to confirm the facilitative role of structural priming in subsequent production, and then to compare the increase in ORC and PRC structures produced in the SCTs from pretest to posttest and delayed posttest. The following is the general profile for the ORC/PRC production over the three phases after removing 2.08% of the missing data at the pretest phase for the control group.

Overall, the proportions of all types of produced structures from the experimental group showed more production of English ORCs than PRCs even though other structures (i.e., SRCs, present participle clauses, reduced PRC, ORC/PRC using the word *who* and ungrammatical structures) were highly responded (Table 4). In the SCT of the pretest phase prior to treatment, English ORCs production was lower for the experimental group (6.87%) than the control group (7.31%) and that of English PRCs was also lower for the experimental group (10.38%) than the control group (12.72%). To statistically verify the difference between two groups at the pretest, a mixed-effects model was fit with ORC/PRC production (1 vs. 0) as the dependent variable. The effects-coded fixed effects predictors were Group (experimental group = −0.5 vs. control group = 0.5), and random intercepts and slopes for subjects and items were also included in the model. No statistically significant difference was found in ORC production between the experimental and control groups (estimate = −0.022, SE = 0.822, *z* = −0.027, *p* = 0.979) or for PRC structures (estimate = 0.087, SE = 0.322, *z* = 0.272, *p* = 0.786). That is, the two groups have no difference in producing English ORCs and PRCs prior to the priming session.

**Table 4:** Percentage (%) of all types of produced sentences in SCT.

Type	Experimental Group	Control Group
ORC	22.22	6.10
ORC using the word *who*	0.10	0.15
PRC	19.59	14.54
PRC using the word *who*	1.22	1.46
Reduced PRC	0.49	1.66
SRC	25.44	42.02
Present Participle Clause	2.78	4.98
Ungrammatical Structure	28.16	29.09

**Figure 6:**
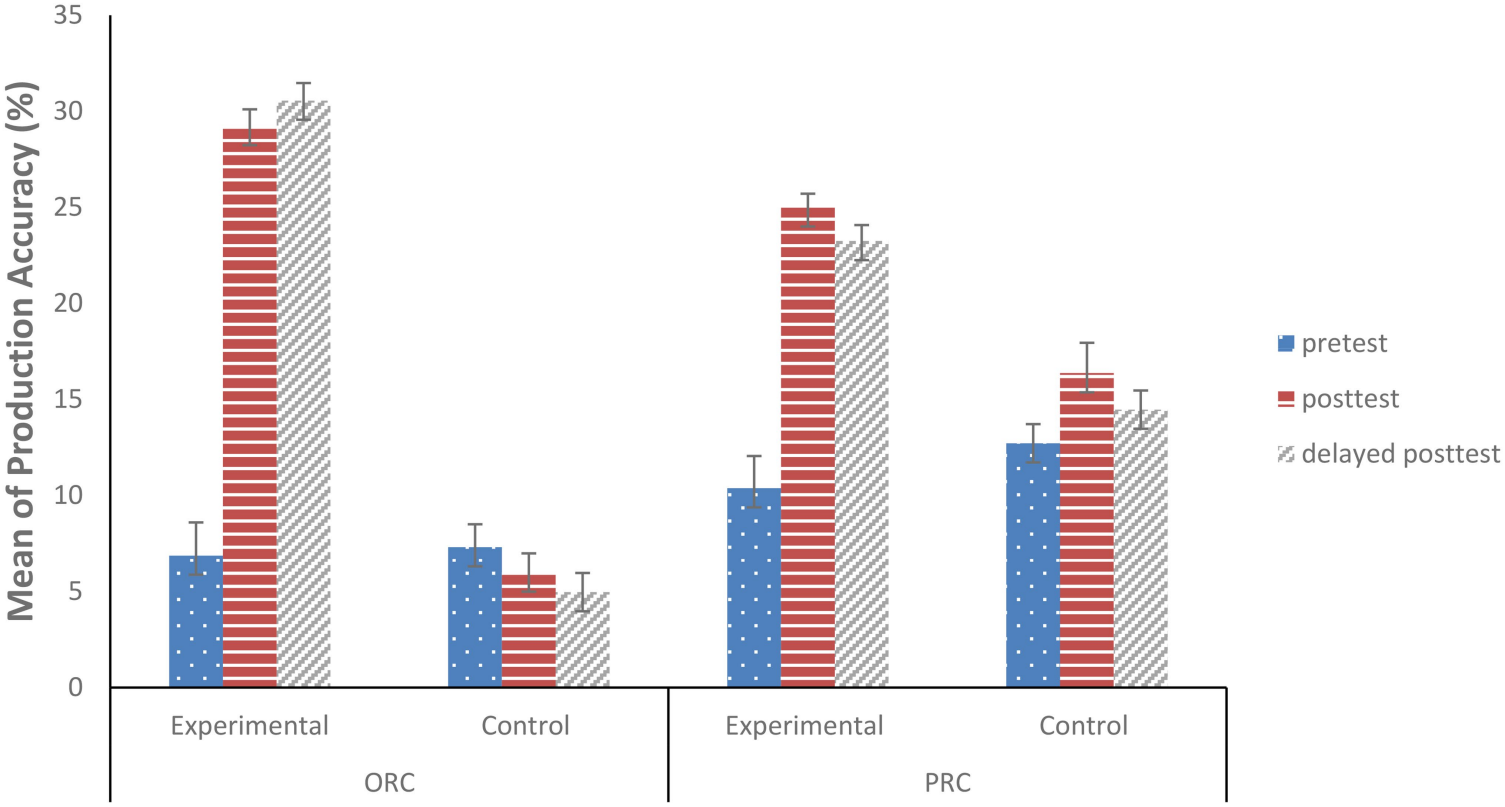
Production of English ORCs and PRCs at Three Phases. Note. Error bars represent 95% CIs for the difference between participant means.

As shown in Figure 6, the experimental group exhibited a sharp increase in production of both ORC and PRC structures from pretest (*M_ORC_* = 6.87%; *M_PRC_* = 10.38%) to posttest (*M_ORC_* = 29.24%; *M_PRC_* = 25%) and delayed posttest phases (*M_ORC_* = 30.56%; *M_PRC_* = 23.25%), while the control group remained relatively stable in their infrequent production of the structures. This also indicates that the performance of the experimental group was sustained through the delayed posttest compared to the control group. However, the increase in ORC production (22.37%) from pretest to posttest was more than for PRC production (14.62%), which indicates that the inverse preference effect exerted an influence on subsequent production.

Similarly, a mixed-effects model was fit with ORC or PRC structures (1 vs. 0) in the SCTs as the dependent variable. All the fixed effects predictors [i.e., effects-coded predictors of Phase: pretest = −0.5 vs. posttest = 0.5 (the remaining variable = 0) and pretest = −0.5 vs. delayed posttest = 0.5 (the remaining variable = 0); Group: experimental = −0.5 vs. control group = 0.5] and their interactions were included in the model. The random effects structure included random intercepts and random slopes for subjects and items.

As shown in Table 5, for ORC production, there were significant main effects for Phase (pretest vs. posttest; estimate = 1.066, SE = 0.193, *z* = 5.534, *p* < 0.001), for Phase (pretest vs. delayed posttest; estimate = 0.745, SE = 0.197, *z* = 3.784, *p* < 0.001), and Group (estimate = −2.114, SE = 0.669, *z* = −3.158, *p* = 0.002), meaning that more ORC structures were produced by the experimental group than the control group, and more in the posttest and delayed posttest than in the pretest. More importantly, interaction effects between Phase (pretest vs. posttest) and Group were found (estimate = −2.171, SE = 0.385, *z* = −5.638, *p* < 0.001), indicating that the experimental group increased more ORC production from pretest to posttest than the control group. Interaction effects between Phase (pretest vs. delayed posttest) and Group were also found (estimate = −2.573, SE = 0.394, *z* = −6.527, *p* < 0.001), the maintenance of the production increase of ORC structures indicating the persistence of the increase in production. For PRC production, the exact same patterns as for ORC production were found. There were significant main effects for Phase (pretest vs. posttest; estimate = 0.754, SE = 0.138, *z* = 5.483, *p* < 0.001), and for Phase (pretest vs. delayed posttest; estimate = 0.398, SE = 0.139, *z* = 2.866, *p* = 0.004) and interactions between Phase (pretest vs. posttest) and Group (estimate = −0.683, SE = 0.275, *z* = −2.485, *p* = 0.013) and between Phase (pretest vs. delayed posttest) and Group (estimate = −0.794, SE = 0.278, *z* = −2.857, *p* = 0.004), even though no significant main effects for Group (estimate = −0.119, SE = 0.444, *z* = −0.268, *p* = 0.789) were found, indicating that the experimental group increased more PRC production from pretest to posttest and from pretest to delayed posttest than the control group. That is, the experimental group produced more PRC structures in the posttest than in the pretest phase, and this persisted into the delayed posttest, which verified the durability of the increase in production.

**Table 5:** Production for ORC and PRC structures at three phases.

	Predictors	Estimate	SE	*z* value	Pr(>|*z*|)
ORC	(intercept)	−4.242	0.402	−10.550	<0.001^***^
	Group (experimental vs. control)	−2.114	0.669	−3.158	0.002^**^
	Phase (pretest vs. posttest)	1.066	0.193	5.534	<0.001^***^
	Phase (pretest vs. delayed posttest)	0.745	0.197	3.784	<0.001^***^
	Group: Phase (pretest vs. posttest)	−2.171	0.385	−5.638	<0.001^***^
	Group: Phase (pretest vs. delayed posttest)	−2.573	0.394	−6.527	<0.001^***^
PRC	(intercept)	−2.808	0.259	−10.845	<0.001^***^
	Group (experimental vs. control)	−0.119	0.444	−0.268	0.789
	Phase (pretest vs. posttest)	0.754	0.138	5.483	<0.001^***^
	Phase (pretest vs. delayed posttest)	0.398	0.139	2.866	0.004^**^
	Group: Phase (pretest vs. posttest)	−0.683	0.275	−2.485	0.013^*^
	Group: Phase (pretest vs. delayed posttest)	−0.794	0.278	−2.857	0.004^**^

^*^p < 0.05, ^**^p < 0.01, ^***^p < 0.001; Final model: ORC/PRC ~ Phase (pretest vs. posttest) * Group + Phase (pretest vs. delayed posttest) * Group + (1|Subject) + (1|Item).

To investigate whether the inverse preference effect extends to subsequent production, another model was run on only the sentences from the experimental group, with ORC/PRC production (1 vs. 0) as the dependent variable. Phase [pretest = −0.5 vs. posttest = 0.5 (the remaining variable = 0) and pretest = −0.5 vs. delayed posttest = 0.5 (the remaining variable = 0)] as the fixed effects predictor was inserted into the model. The random effects structure included random intercepts and random slopes for subjects and items. A statistically significant main effect was found for Phase (pretest vs. delayed posttest; estimate = 0.858, SE = 0.337, *z* = 2.545, *p* = 0.011). This indicates that the increase in ORC production from pretest to delayed posttest was stronger than that for PRCs. The inverse preference effect therefore extended to subsequent production.

To summarize, the experimental group produced more ORC and PRC structures than the control group in subsequent posttest phases, and the production endured into the delayed posttest. That is, structural priming did promote the subsequent production of the target structures. Additionally, the increased production for ORCs was stronger than that for PRCs. Therefore, inverse preference effects did extend to subsequent production of target structures.

## Discussion

The present study investigated the inverse preference effect on Chinese learners’ grammaticality judgments and production of English ORC and PRC structures. Our study first confirmed the occurrence of structural priming in L2 learners’ immediate primed production of English ORCs and PRCs, as learners were more likely to subsequently produce ORC structures after ORC primes in the priming task than after PRC primes; likewise, learners were prone to produce more PRC structures after PRC prompts than after ORC prompts. The findings also revealed that structural priming promoted subsequent grammaticality judgments for English ORCs, but not so for English PRCs. Finally, for priming effects in subsequent production, the findings showed that structural priming promoted subsequent production of ORC and PRC structures after priming along with long-lasting effects, and the inverse preference effects extended into posttest sessions.

### Inverse Preference Effects and Their Impact on L2 Priming

For the first research question, we found structural priming in L2 learners’ immediate primed production of English ORCs and PRCs. These findings are consistent with previous L2 priming studies, which also found structural priming across a variety of target structures (e.g., Kim and McDonough, 2008; Shin and Christianson, 2012; McDonough and Fulga, 2015). For structural complexity in priming effects, our research findings indicate inverse preference effects in structural priming, as the low frequency target structures (i.e., English ORCs) are more successfully primed than the alternative high frequency structure (i.e., English PRCs). The inverse preference effect in immediate primed production may be due to the informational value of prime sentences (Scheepers, 2003). The familiarity of a prime can weaken the strength of the information provided (e.g., the syntactic structure). Conversely, a less favored structure is more likely to be primed since the lack of structural information could highlight its particularity and then lead to more information being primed accordingly.

Our findings are also in line with accounts of the implicit learning mechanism of structural priming (e.g., Bock and Griffin, 2000; Chang et al., 2006) in that structural priming effects result from speakers’ long-term implicit or unconscious adaptation of syntactic knowledge. The learning mechanism behind structural priming assumes that the relevant type of learning is implicit and procedural, as it does not depend on specific intentions, require memory effort, or require explicit attention to the particular form of a priming sentence (Bock, 1986; Bock et al., 1992). It emphasizes the unconscious, automatic character of syntactic repetition and syntactic features, and their abstractness and duration, all of which are the foundation of learning processes. Specifically, Chang et al.’s (2006) account of error-based implicit learning assumes structural priming arises from speakers’ adaptation of syntactic knowledge after their failure on upcoming word predictions. The low-frequency structure is less likely to be expected and thus yields higher prediction errors than its high frequency counterpart. Higher prediction errors correspondingly yield greater weight changes, leading to greater structural bias toward low frequency structures, that is, inverse preference effects.

### Inverse Preference Effects and Their Impact on L2 Learning

Prior to the priming, we first checked the structural bias for English ORCs rather than PRCs and found the low frequency of English ORCs than PRCs in the pre-priming production. With these inverse preference effects, we found that structural priming promoted subsequent grammaticality judgments for English ORCs, but not so for English PRCs. Since grammaticality judgments concern receptive syntactic knowledge of target structures, the facilitative role of structural priming in subsequent judgments was in line with Kim and McDonough’s (2016) findings showing a priming effect promoting L2 learners’ subsequent comprehension of English passives in picture matching tasks. However, with the same task type, i.e., a grammaticality judgment task, our results are somewhat different from those of Shin and Christianson (2012), which only found a marginally significant improvement between pretest and posttest for dative and phrasal-verb structures among L2 Korean learners of English. They attributed the weak effects for the grammaticality judgment task and subsequent L2 learning after priming to the difference between receptive and productive knowledge, and believed that the priming task was mainly designed to improve productive knowledge *via* production rather than to promote improvement of receptive knowledge. The discrepancy between Shin and Christianson (2012) and the present study may well be due to the difference in structural complexity (simple structure vs. complex structure). There seem to be no difficulties for learners with years of learning experience to comprehend the meaning and judge receptive knowledge of English datives and phrasal verb structures. On the other hand, considering the difficulties of low-to-intermediate Chinese learners in learning the complex structures of English relative clauses, it is not particularly surprising that structural priming facilitates subsequent grammaticality judgments to a statistically significant level.

For the null effect for subsequent grammaticality judgment accuracy for English PRCs, one potential explanation is that the development of L2 learners’ abstract syntactic knowledge of ORCs and PRCs is inconsistent. It was found that participants could accurately judge nearly 70% of PRC structures at pretest, suggesting that they might have had complete knowledge of the morphological, semantic, and discourse constraints to attain a conceptual understanding of English PRCs prior to the experiment. A ceiling effect for PRC structures hindered any further improvement in their performance *via* the priming task. Conversely, a lack of prior knowledge of ORC structure led to incomplete development of abstract syntactic information and then a lower degree of judgment accuracy for English ORCs at pretest. Their subsequent improvement in judgment accuracy was thus salient, in that the priming task offered repetitive input and output of ORC structures, which ultimately enhanced their conceptual understanding and competence. Another possible concern comes from structural complexity. English PRCs are more preferred and accessible for comprehension, since they resemble the structure of English passives but with the patient and predicate separated by a relativizer (*that*). The ORC structure is less preferred and more difficult to process, since the word order differs greatly between the structure of English actives (e.g., *a cat chased a dog*) and the structure of English ORCs (e.g., *a dog that a cat chased*). The apparent word order distinction between English ORCs and English actives posed challenges to the L2 learners in structural understanding and in providing accurate responses accordingly.

For priming effects in subsequent production, the findings showed that structural priming promoted subsequent production of ORC and PRC structures after priming with long-lasting effects, and the inverse preference effects were also found from the priming to the posttest sessions. The importance of structural priming in promoting subsequent production of ORC and PRC structures echoes previous L2 priming studies that have reported a positive contribution of structural priming to subsequent production of target structures (McDonough and Mackey, 2008; McDonough and Kim, 2009; Shin and Christianson, 2012; McDonough et al., 2015). This is possibly attributable to the opportunity that structural priming provides for L2 learners for repetitive input and output of target structures. In the light of the cognitive load of complex structures, for L2 learners, it is preferable to choose simpler structures that they are more familiar with in oral production than under-developed complex structures. However, as the error-based implicit learning account stated, L2 learners previously predict about the upcoming sentence structures (e.g., simple structures) and then they experience a prediction error when their predictions are not met. These prediction error causes structural priming which helps learners update their implicit expectations by providing a greater quantity of ORCs/PRCs as priming prompts and contributes to their subsequent production of target structures.

The inverse preference effect in subsequent production, on the one hand, may be attributable to the structural frequency of English ORCs and PRCs in both L1 and L2. From the perspective of L1, there is a structural difference of relative clause between L1 Chinese and L2 English. English ORCs/PRCs are head-initial that differ from head-final structures in Chinese relative clauses. The null L1 equivalence therefore leads to learning obstacles of both English ORCs/PRCs for L2 learners, especially lower-intermediate learners. Furthermore, English PRC is more likely to be produced than ORC, noted from previous L1 studies (Roland et al., 2007; Gennari and MacDonald, 2009; Gennari et al., 2012) and our pre-priming L2 results. The error-based implicit learning account therefore suggests the less expected the structure is, the greater the prediction error and priming effect will be, leading to a long-lasting learning effect. With regard to L2 proficiency, Montero-Melis and Jaeger (2020) found that less proficient speakers exhibited the inverse preference effect based on the unexpectedness in L1, but more proficient ones showed the inverse preference effect based on the unexpectedness in L2. Our study found that structural frequency is driven by a mixture of both L1 and L2, especially for complex structures. Concerning the structural difference of ORCs vs. PRCs in L1 and their structural frequency in L2, it is understandable that English ORCs is less expected but stronger priming and learning effect than English PRCs, presenting the inverse preference effect in subsequent production. This also supports the persistence of inverse preference effect in L2 learning.

## Conclusion

The current study sheds light on the importance of structural priming in L2 grammaticality judgments and the production of complex syntactic structures. Structural priming facilitated L2 learners’ immediate priming and subsequent grammaticality judgment and production of English ORC and PRC structures with long-lasting effects. For the inverse preference effects, the findings confirmed the presence of inverse preference effects during the immediate priming session, and the effect did extend to subsequent grammaticality judgments and production; the magnitude of priming effects in promoting L2 judgments of English ORC structures was stronger than for English PRCs. This therefore suggests that structural priming, like practice, is beneficial for L2 learners’ long-term grammaticality judgments and production of complex structures, yet its effect can be affected by the complexity of target structures and structural frequency of both L1 and L2.

## Data Availability Statement

The raw data supporting the conclusions of this article will be made available by the authors, without undue reservation.

## Ethics Statement

The studies involving human participants were reviewed and approved by The Hong Kong Polytechnic University. The patients/participants provided their written informed consent to participate in this study.

## Author Contributions

RW led the study, conceived the original idea, collected and analyzed the data, and wrote the first draft of the manuscript. SK supervised RW’s work, provided financial support, and edited the manuscript. JS contributed to data analysis and manuscript editing. All authors contributed to the article and approved the submitted version.

## Funding

This work was supported by the Doctoral Teachers’ Scientific Research Support Project of Jiangsu Normal University (grant no. 19XFRX001).

## Conflict of Interest

The authors declare that the research was conducted in the absence of any commercial or financial relationships that could be construed as a potential conflict of interest.

## Publisher’s Note

All claims expressed in this article are solely those of the authors and do not necessarily represent those of their affiliated organizations, or those of the publisher, the editors and the reviewers. Any product that may be evaluated in this article, or claim that may be made by its manufacturer, is not guaranteed or endorsed by the publisher.

## Footnotes

^1^According to Hulstijn and Graaff (1994), structures can be divided into complex and simple in which the complex structures have multiple components and abstract knowledge, while the simple structures do not. Therefore, complex structures (i.e., complex sentences with one dependent/subordinate clause joined to an independent/main clause) involve different lexical content and abstract syntax, whereas simple structures (i.e., simple sentences with only one independent/main clause) include simple lexical items and less abstract syntax.

^2^This refers to experimental designs in which all the primes occur before all the targets, i.e., repetitive exposure to prime sentences is followed by repetitive production of target sentences.

^3^Likewise, based on the first author’s long experience of teaching English to Chinese learners in China, Chinese learners have more difficulties in producing ORCs than PRCs, especially when both the head noun in the matrix and the noun in the clause are animate in nature.

^4^Schulz, B. (2006). *Wh-scope marking in English interlanguage grammars: Transfer and processing effects on the second language acquisition of complex wh-questions* [Unpublished doctoral dissertation]. University of Hawai’i, Honolulu, US.

^5^In this study, “ORC/PRC production” refers to grammatically correct completions of English ORCs and PRCs, which is a much narrower concept and usage than a general term of “production,” in order to contrast with a comprehension aspect measured by a grammatical judgment task in the study.

^6^Note that here the two nouns have no semantic association, such as “doctor-nurse” or “mother-baby,” because semantic closeness can influence the structural priming effect (Cleland and Pickering, 2003).
