# Supplementary material for: Structural Priming and Inverse Preference Effects in L2 Grammaticality Judgment and Production of English Relative Clauses
Source: Front Psychol. 2022 Jun 22;13:845691. doi: 10.3389/fpsyg.2022.845691 (PMC9258510; doi:10.3389/fpsyg.2022.845691)
Supplement: Supplementary file 1 [file Data_Sheet_1.pdf]

Appendix 1 Materials for Grammaticality Judgment Task (GJT)

Pretest (*N* = 20)

The woman met the teacher that she missed him every second.
The partner that was fired the boss polished the manuscript.
The man served the female customer that he noticed for a while.
The manager fired the interviewee that learnt everything slowly.
The female worker stopped the manager that she watched him.
The daughter that was caught by the sailor lost a single deal.
The husband that the brother trusted bought an expensive gift.
The cashier that he blundered the account reformulated his utterance.
The neighbor found the friend that was annoyed by the monkey.
The engineer that was understood the nurse cheated the citizen.
The woman noticed the manager that she missed him at Christmas.
The postman disliked the cartoonist that he screwed up everything.
The daughter stopped the nurse that she consulted after the operation.
The neighbor that was hurt by the sailor finally found his parents.
The engineer that the soldier contacted fixed the disk drive by himself.
The teacher that was met the friend had 21 years' experience.
The fireman that rescued the banker controlled the entire situation.
The visitor that was noticed the man got lost in the forest for a day.
The female partner missed the chef that she always served him.
The worker caught the citizen that was watched by the enemy.

Posttest (*N* = 20)

The student that the colleague needed died in a car accident a week ago.
The speaker approached the chairwoman that she was influenced by him.
The member that the cowboy left him forgot an unusual thing yesterday.
The manager fired the interviewee that learnt everything slowly.
The patient followed the sister that he remembered for a long time.
The lawyer that was hit the editor has run the firm well for a year.
The artist that the audience visited him moved to another city in the US.
The postman disliked the cartoonist that he screwed up everything.
The star that was supported by the dentist never wrote anything after that day.
The cashier that he blundered the account reformulated his utterance.
The host talked to the actor that was seldom forgotten by others in the town.
The star that the audience needed decided to change his clothing style.
The member that was forgotten his colleague was no longer with the company.
The fireman that rescued the banker controlled the entire situation.
The speaker that the student followed him went home immediately.
The graduate remembered the editor that was hit by the patient in the hospital.

The actress visited the lawyer that she just knew last week.
The guy that was taught by the scientist entered a top university in China.
The son that the housekeeper remembered him refused to go back home.
The sister influenced the boy that he was raised by her since their parents died.

Delayed Posttest (*N* = 20)

The model that the officer helped promised to sign the contract on time.
The manager chose the author that was already accepted by the public.
The dealer that the wife called him suffered a horrible night.
The manager fired the interviewee that learnt everything slowly.
The runner believed the director that he respected at first sight.
The secretary killed the owner that attacked by the enemy in the war.
The postman disliked the cartoonist that he screwed up everything.
The writer that was recognized by the guest never forgave his delay.
The lady attacked the driver that she disliked him for her whole life.
The assistant that was disliked the employer welcomed the guest for a day.
The fireman that rescued the banker controlled the entire situation.
The farmer that the doctor assisted sent a thank-you letter yesterday.
The police officer that was supported by the director was very popular in the city.
The cashier that he blundered the account reformulated his utterance.
The secretary that the guest believed him told a lie in front of his' son.
The driver attacked the lady that controlled by the police after the accident.
The officer chose the mayor that he supported without any hesitation.
The ambassador called the minister that was respected by the citizens.
The wife accepted the assistant that she had believed him for a long time.
The trainee that was recognized the employer dropped out in June.

Appendix 2 Materials for Oral Sentence Completion Task (SCT)

Pretest (*N* = 12)

Here is ______________________________. (meet, customer, teacher)
Here is ______________________________. (see, man, woman)
Here is ______________________________. (find, manager, brother)
Here is ______________________________. (fire, husband, worker)
Here is ______________________________. (recommend, sailor, chef)
Here is ______________________________. (love, daughter, nurse)
Here is ______________________________. (trust, partner, singer)
Here is ______________________________. (notice, citizen, researcher)
Here is ______________________________. (hurt, tourist, enemy)
Here is ______________________________. (miss, grandfather, soldier)
Here is ______________________________. (contact, engineer, visitor)
Here is ______________________________. (consult, author, hero)

Posttest (*N* = 12)

Here is ______________________________. (need, student, translator)
Here is ______________________________. (leave, cowboy, patient)
Here is ______________________________. (follow, son, colleague)
Here is ______________________________. (know, star, adult)
Here is ______________________________. (visit, housekeeper, sister)
Here is ______________________________. (remember, audience, artist)
Here is ______________________________. (approach, speaker, guardian)
Here is ______________________________. (teach, hostess, passenger)
Here is ______________________________. (raise, dentist, editor)
Here is ______________________________. (forget, lawyer, graduate)
Here is ______________________________. (influence, chairman, actor)
Here is ______________________________. (hit, talent, pilot)

Delayed posttest (*N* = 12)

Here is ______________________________. (call, father, model)
Here is ______________________________. (help, wife, employee)
Here is ______________________________. (believe, director, doctor)
Here is ______________________________. (choose, officer, dealer)
Here is ______________________________. (reject, lady, writer)
Here is ______________________________. (attack, secretary, driver)
Here is ______________________________. (respect, owner, guest)
Here is ______________________________. (dislike, boy, musician)
Here is ______________________________. (rescue, runner, waitress)
Here is ______________________________. (support, police, mayor)

Here is ____________________________. (assist, dancer, athlete)
Here is ____________________________. (persuade, leader, gentleman)

Appendix 3 Materials for the Priming Task

Critical Stimuli

1. Here is the officer that the model called.
   Here is the officer that was called by the model.
2. Here is the dealer that was requested by the sponsor.
   Here is the dealer that the sponsor requested.
3. Here is the father that the doctor helped.
   Here is the father that was helped by the doctor.
4. Here is the scholar that was supported by the teenager.
   Here is the scholar that the teenager supported.
5. Here is the employee that the wife believed.
   Here is the employee that was believed by the wife.
6. Here is the nephew that was welcomed by the grandma.
   Here is the nephew that the grandma welcomed.
7. Here is the child that the neighbor disliked.
   Here is the child that was disliked by the neighbor.
8. Here is the poet that was selected by the cousin.
   Here is the poet that the cousin selected.
9. Here is the boy that the writer liked.
   Here is the boy that was liked by the writer.
10. Here is the uncle that was promoted by the mayor.
    Here is the uncle that the mayor promoted.
11. Here is the driver that the secretary chose.
    Here is the driver that was chosen by the secretary.
12. Here is the lover that was aided by the athlete.
    Here is the lover that the athlete aided.
13. Here is the owner that the lady killed.
    Here is the owner that was killed by the lady.
14. Here is the volunteer that was interviewed by the journalist.
    Here is the volunteer that the journalist interviewed.
15. Here is the leader that the monitor recorded.
    Here is the leader that was recorded by the monitor.
16. Here is the granddaughter that was complained by the grandson.
    Here is the granddaughter that the grandson complained.
17. Here is the minister that the guest attacked.
    Here is the minister that was attacked by the guest.
18. Here is the trainer that was employed by the master.

Here is the trainer that the master employed.

19. Here is the builder that the farmer feared.
    Here is the builder that was feared by the farmer.
20. Here is the baker that was rejected by the cook.
    Here is the baker that the cook rejected.
21. Here is the employer that the assistant respected.
    Here is the employer that was respected by the assistant.
22. Here is the deliverer that was appointed by the resident.
    Here is the deliverer that the resident appointed.
23. Here is the politician that the producer recognized.
    Here is the politician that was recognized by the producer.
24. Here is the manufacturer that was educated by the gentleman.
    Here is the manufacturer that the gentleman educated.
25. Here is the servant that the immigrant refused.
    Here is the servant that was refused by the immigrant.
26. Here is the slave that was persuaded by the consultant.
    Here is the slave that the consultant persuaded.
27. Here is the scientist that the waitress ignored.
    Here is the scientist that was ignored by the waitress.
28. Here is the dancer that was evaluated by the instructor.
    Here is the dancer that the instructor evaluated.
29. Here is the victim that the guard abandoned.
    Here is the victim that was abandoned by the guard.
30. Here is the monk that was avoided by the guy.
    Here is the monk that the guy avoided.
31. Here is the opponent that the angel assisted.
    Here is the opponent that was assisted by the angel.
32. Here is the smoker that was rescued by the runner.
    Here is the smoker that the runner rescued.
33. Here is the analyst that the police hired.
    Here is the analyst that was hired by the police.
34. Here is the jumper that was greeted by the helper.
    Here is the jumper that the helper greeted.
35. Here is the evil that the god judged.
    Here is the evil that was judged by the god.
36. Here is the madam that was emailed by the queen.
    Here is the madam that the queen emailed.

Fillers

1. The lion lied under the tree.
2. The rabbit ran as fast as the dog.
3. The story seemed interesting.
4. Here came the ambulance.

5. She smiled unnaturally.
6. The story was not as interesting as that one.
7. The windows were broken.
8. Under the tree was lying a wounded tiger.
9. The airplane arrived at Beijing on time.
10. My mother always got up earlier than me.
11. The building remained there.
12. There stood a dog before him.
13. The bird sang well on the branch.
14. Lily was the taller of the twins.
15. The vet looked tired after the operation.
16. Such was the story he told me.
17. The HR spoke at the meeting yesterday.
18. The weather of Kunming is better than that of Wuhan.
19. The postman was a rogue.
20. Away went the clown.
21. The birds flew freely in the sky.
22. The comedian drank more beer than me.
23. The silk felt soft and smooth.
24. At the foot of the hill was a beautiful lake.
25. Snow White danced with the music.
26. The technician was two years older than the driller.
27. It sounded like a good idea.
28. Seated on the ground was a group of young people.
29. The teddy bear lay on the bed.
30. The box was heavier than that one.
31. The dish tasted delicious.
32. Lying on the floor was a cowboy.
33. The watch stopped.
34. The weather became hotter and hotter.
35. The beggar became mad after that.
36. Then came the hour we had been looking forward to.
37. The meeting began at eight thirty.
38. Health was more important than wealth.
39. The milk soon turned sour overnight.
40. In came the barber.
41. The accident happened yesterday evening.
42. The quicker you get ready, the sooner we’ll be able to leave.
43. The waitress had grown thinner and thinner.
44. There existed different opinions on this question.
45. Tom ran fast enough to win the game.
46. This was the most beautiful scene that I have seen.
47. The witch went pale at the news.
48. Walking at the head of the line was our mentor.

49. The detective sat silently to observe the suspect.
50. It took more hours to go there by train than by air.
51. His story proved false.
52. Present at the meeting were the elite in the field.
53. The maid listened but did not hear anything.
54. The ship came later than usual.
55. My intuition turned out to be correct.
56. Standing beside the desk was the fireman.
57. The trainee swam a few laps as a warm-up during the practices.
58. The internet gave us the latest news.
59. The winner looked excited after the game.
60. Up went the arrow into the air.
61. She cried in the corner after the performance.
62. English became more and more important in our daily life.
63. His hair turned grey in a few weeks.
64. East of the lake were two cities.
65. The door opened automatically.
66. That one was the most expensive car in the store.
67. Everybody felt content after the reform.
68. There went the postman.
69. The headache only occurred at night.
70. I had nothing further to say on the subject.
71. This kind of cloth felt very soft.
72. Out rushed the beggar.

Appendix 4 Target Elicitation During Priming Treatment

1. Here is ______________________. (find, teacher, man)
2. Here is ______________________. (fire, woman, customer)
3. Here is ______________________. (meet, worker, manager)
4. Here is ______________________. (see, chef, husband)
5. Here is ______________________. (watch, daughter, partner)
6. Here is ______________________. (stop, brother, sailor)
7. Here is ______________________. (control, author, director)
8. Here is ______________________. (love, engineer, nurse)
9. Here is ______________________. (annoy, hero, visitor)
10. Here is ______________________. (miss, tourist, citizen)
11. Here is ______________________. (notice, researcher, grandfather)
12. Here is ______________________. (catch, enemy, soldier)
13. Here is ______________________. (introduce, winner, photographer)
14. Here is ______________________. (admire, friend, musician)
15. Here is ______________________. (promise, pupil, mother)
16. Here is ______________________. (contact, prisoner, terrorist)
17. Here is ______________________. (protect, designer, singer)
18. Here is ______________________. (recommend, girl, advisor)
19. Here is ______________________. (invite, operator, shareholder)
20. Here is ______________________. (trust, historian, reader)
21. Here is ______________________. (warn, publisher, buyer)
22. Here is ______________________. (hate, reporter, boss)
23. Here is ______________________. (blame, president, professor)
24. Here is ______________________. (hurt, listener, host)
25. Here is ______________________. (suspect, competitor, holder)
26. Here is ______________________. (advise, person, stranger)
27. Here is ______________________. (kiss, baby, kid)
28. Here is ______________________. (doubt, expert, fan)
29. Here is ______________________. (insult, governor, specialist)
30. Here is ______________________. (criticize, tutor, housewife)
31. Here is ______________________. (praise, babysitter, violinist)
32. Here is ______________________. (consult, painter, coach)
33. Here is ______________________. (advocate, waiter, cleaner)
34. Here is ______________________. (embrace, swimmer, guide)
35. Here is ______________________. (bite, salesman, princess)
36. Here is ______________________. (chase, prince, king)
